# Chemerin and PEDF Are Metaflammation-Related Biomarkers of Disease Activity and Obesity in Rheumatoid Arthritis

**DOI:** 10.3389/fmed.2018.00207

**Published:** 2018-08-03

**Authors:** Barbara Tolusso, Maria Rita Gigante, Stefano Alivernini, Luca Petricca, Anna Laura Fedele, Clara Di Mario, Barbara Aquilanti, Maria Rosaria Magurano, Gianfranco Ferraccioli, Elisa Gremese

**Affiliations:** ^1^Division of Rheumatology, Fondazione Policlinico Universitario A. Gemelli, IRCCS, Catholic University of the Sacred Heart, Rome, Italy; ^2^Service of Dietary and Human Nutrition, Fondazione Policlinico Universitario A. Gemelli, IRCCS, Catholic University of the Sacred Heart, Rome, Italy; ^3^Service of Psychology and Psychotherapy, Fondazione Policlinico Universitario A. Gemelli, IRCCS, Catholic University of the Sacred Heart, Rome, Italy

**Keywords:** Rheumatoid arthritis, adipose tissue, Chemerin, PEDF, metaflammation

## Abstract

**Objective:** Obesity is a risk factor for Rheumatoid Arthritis (RA) being associated to low grade inflammation. This study aimed to determine whether PEDF and Chemerin are biomarkers of inflammation related to fat accumulation in RA and to investigate whether weight loss associates with clinical disease improvement through the modification of fat-related biomarkers in overweight/obese RA with low-moderate disease.

**Participants and Methods:** Two-hundred and thirty RA patients were enrolled, of whom 176 at disease onset treated according to a treat-to-target strategy (T2T) and 54 overweight/obese RA in stable therapy and low-moderate disease activity. Gene expression of adipokines, interleukin-6 and their receptors were examined in adipose tissue from obese RA. Obese RA with low-moderate disease activity underwent low-calories diet aiming to Body Mass Index (BMI) reduction >5%, maintaining RA therapy unchanged. Chemerin, PEDF and Interleukin-6 plasma values were assessed by ELISA and disease activity was evaluated.

**Results:** At RA onset, PEDF and Chemerin plasma values correlated with BMI (*p* < 0.001) but only Chemerin plasma values correlated with disease activity (*p* < 0.001). After adopting a T2T strategy, Chemerin arose as an independent factor associated with remission in early RA [OR(95%CIs):0.49(0.25–0.97)]. Moreover, after low-calories diet, RA with low-moderate disease activity reaching BMI reduction ≥5% (62.6%) at 6 months had significant decrease of PEDF (*p* < 0.05) and Chemerin (*p* < 0.05) plasma values, in parallel with the improvement in disease activity.

**Conclusions:** PEDF and Chemerin arose as biomarkers of obesity and metaflammation respectively, providing a link between chronic inflammation and excess of body weight in RA. Therefore, BMI reduction of at least 5% in obese RA allowed better disease control without modifying RA treatment.

## Introduction

Obesity is a risk factor for autoimmune diseases as Rheumatoid Arthritis (RA) (1–3) since adipose tissue releases adipokines able to create a low grade inflammatory environment (4–8). However, the link between obesity and RA is not fully clarified since there are no parameters available at the moment to understand the inflammation related to fat accumulation and the behavior of fat-inflammation under weight loss.

Among adipokines, pigment epithelium derived factor (PEDF) is one of the most abundant proteins secreted during adipocytes maturation directly linked to central obesity (9–12). Recently, Chemerin has emerged as a key adipokine involved in immune response. Overweight and obese healthy subjects show higher Chemerin values than normal weight, whose plasma values decrease after diet (13, 14). Despite Chemerin was found in inflamed tissues and biological fluids of RA, promoting synovial fibroblasts hyperplasia (15–17), it is not completely known whether Chemerin is associated with systemic inflammation or with adipose tissue activation in RA.

To date, there are only few data regarding the influence of body fat on RA disease activity suggesting that obesity associates with disease outcomes (18, 19). Therefore, the aims of our study were: (i) to dissect whether PEDF and Chemerin are biomarkers of obesity and fat-related inflammation (metaflammation) in RA, influencing disease activity at RA onset. Moreover, since overweight/obesity status influences the chance of good clinical outcome achievement in RA, (ii) to evaluate whether in obese RA patients with low-moderate disease activity, weight loss, mirrored by fat-related biomarkers, without RA treatment change might improve the clinical outcome.

## Subjects and methods

### Participants

#### Study population-1

To verify the value and role of biomarkers in deciphering the metaflammation and inflammation course, 176 consecutive early-RA (ERA) patients, fulfilling the 2010 American College of Rheumatology criteria for RA (20), were studied at disease onset. Subjects with symptoms duration < 3 months were defined as “very early RA” (VERA) (21). All ERA were naïve to conventional Disease-Modifying Anti-Rheumatic Drugs (cDMARDs) and/or biological (bDMARDs) and were followed according to the “treat-to-target strategy” (T2T) (22). For each RA patient, the ACR/European League Against Rheumatism core data set and disease activity scores (DAS/SDAI) were recorded (23).

BMI at study entry and during follow-up was recorded for each patient (24). A comparison of sex and age matched healthy group (*n* = 30) was enrolled.

The study was approved by the local Ethical Committee and all participants gave their signed informed consent.

#### Study population-2

Fifty-four overweight/obese RA patients, with moderate disease activity (DAS < 3.7) for at least 12 weeks despite cDMARDs and/or bDMARDs treatment were enrolled. Each patient underwent a dietary intervention (1,000–1,200 kcal/day) (25) for at least 6 months under nutritionist/psychologist supervision aiming to a BMI reduction >5% within 6 months without RA therapy changes (26, 27). Since in any trial of diet induced weight loss, a mean of 15% of patients do not succeed in obtaining the 5% BMI reduction, we decided to use the unsuccessful subset of patients as an internal control group (28).

#### White adipose tissue (WAT) biopsy and gene expression by qPCR in WAT

Twenty-seven of the obese RA patients underwent abdominal subcutaneous adipose tissue biopsy using an aspiration technique. Ten age and sex matched obese osteoarthritis (OA) patients were enrolled as controls.

Total RNA was isolated from adipose tissue in TRIzol® (Invitrogen, Carlsbad, CA) according to the manufacturer protocol. iScript™ cDNA Synthesis Kit (Bio Rad Laboratories, Hercules, CA) was used for cDNA preparation. FastStart Universal Probe Master (Roche Diagnostics, Germany) was used for qPCR. Primers for human *RARRES2 (Chemerin), CMKLR1 (Chemerin receptor23), SERPINF1 (PEDF), IL6, IL6-R*, and *GAPDH*, as endogenous control, were used (Roche Diagnostics, Germany). The relative expression fold change of each target gene was calculated using the comparative CT method with 2^∧^−ΔΔC_T_ equation.

#### Chemerin, PEDF, sIL-6R, and IL-6 plasma values assessment

For each enrolled RA patient, peripheral blood samples were collected, immediately processed and stored at −80°C until analysis. IL-6, sIL-6R, PEDF and Chemerin plasma values were measured by ELISA (R&D Systems, UK). The sensitivity of the test was 0.7 pg/ml for IL-6, 6.5 pg/ml for sIL-6R, 0.045 ng/ml for PEDF and 4.13 pg/ml for Chemerin.

### Statistical analysis

Statistical analysis was performed using SPSS (SPSS version 20.0, Chicago, IL, USA) and Graph-Pad Prism statistical software (San Diego, CA, USA). Categorical and quantitative variables were recorded as frequencies, percentages, mean ± Standard Deviation (SD). The non-parametric Mann-Whitney *U*-test and the χ^2^ test were used, as appropriate. The Spearman rank correlation was used to evaluate the relationship between fat-related parameters and inflammatory and clinical parameters and the Wilcoxon test was used to compare the clinical parameters and the soluble biomarkers during follow-up. A receiver operating characteristic (ROC) analysis of the adipokines related to BMI was performed.

Sample size for Study population-2 was calculated by G^*^Power 3.1 software (29) using as reference the successful rate of outcome achievement (BMI reduction > 5%) in the general population (27), obtaining a minimum sample size of 41 individuals with a study power of 82.2% and α = 0.04.

A multivariate logistic regression model, in which “DAS or SDAI remission at 6 or 12 month follow-up visit” were the dependent variables to be explained, was performed and results were expressed as the odds ratio (OR) and 95% confidence interval (95%CI). Statistical significance was defined as *p* < 0.05.

## Results

### Overweight and obesity status, mirrored by adipokines milieu, is related to disease activity of RA patients at onset

**Figure 1:**
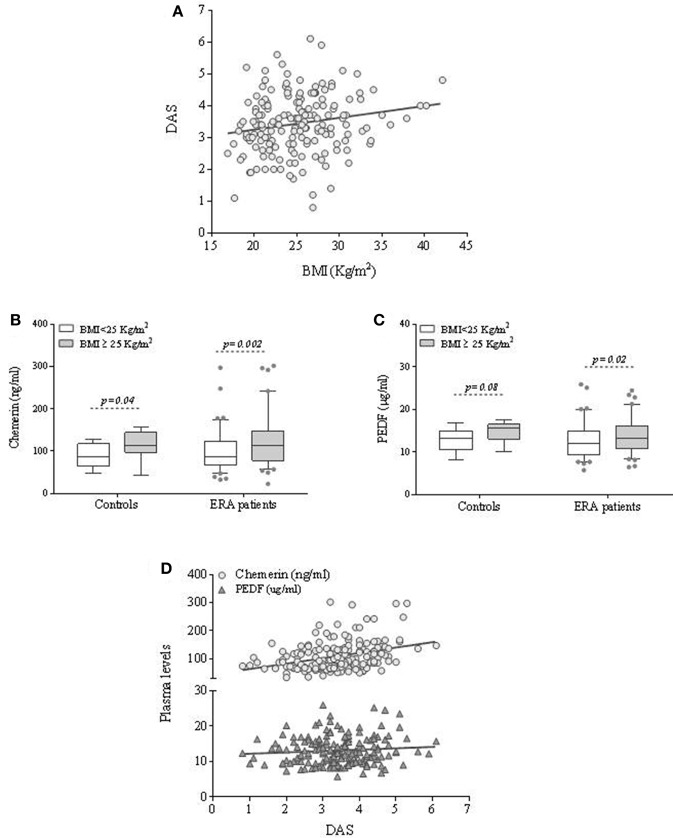
Body mass index, PEDF and Chemerin plasma values are reciprocally related and associated to disease activity in ERA patients at disease onset. **(A)** Correlation between disease activity and BMI in ERA patients at baseline. **(B,C)** Plasma adipokines (PEDF and Chemerin) values in ERA patients at diagnosis and controls based on the BMI category. **(D)** Correlation between disease activity and PEDF and Chemerin plasma values in ERA patients at baseline.

To dissect the contribution of fat mass on clinical phenotype at RA onset, 176 ERA patients were enrolled as investigational cohort (Study population-1). Baseline demographic and clinical characteristics are shown in Table 1. Overweight and obese ERA patients showed higher disease activity than normal weight ones (Table 1) (Figure 1A) (Supplemental Table 1).

**Table 1:** Demographic, immunological and clinical characteristics of Study populations 1 and 2.

	**Study population-1**					**Study population-2**
	**All**	**BMI ≥ 25 Kg/m^2^**	**BMI < 25 Kg/m^2^**	***p*-value**	*p*-value*	
*N*	176	89	87			54
Age, years	56 ± 15	58 ± 13	53 ± 16	0.02		57 ± 12
Symptom^#^/Disease^##^'s duration, months^#^/years^##^	5.6 ± 3.5	5.2 ± 3.2	6.1 ± 3.6	0.09		5.9 ± 3.6
Sex, n. female (%)	135 (76.7)	63 (70.8)	72 (82.8)	0.06		46 (85.2)
BMI, Kg/m^2^	25.3 ± 4.8	–	–	–		35.2 ± 4.1
Overweight, n(%)	63 (35.8)	–	–	–		4 (7.4)
Obesity, n(%)	26 (14.8)	–	–	–		50 (92.6)
ACPA positivity, n(%)	104 (59.1)	54 (60.7)	50 (57.5)	0.67		46 (48.1)
RF-IgM positivity, n(%)	84 (47.7)	46 (51.7)	39 (44.8)	0.36		22 (40.7)
RF-IgA positivity, n(%)	51 (29.0)	30 (33.7)	21 (24.1)	0.16		14 (25.9)
ESR, mm/1^∧^hour	41.4 ± 28.7	44.3 ± 27.7	38.5 ± 29.5	0.07	0.49	35.3 ± 22.9
CRP, mg/l	21.3 ± 31.5	25.7 ± 36.4	16.8 ± 25.0	0.02	0.24	9.5 ± 10.8
IL6, pg/ml	21.7 ± 42.6	26.7 ± 43.1	13.8 ± 21.3	0.01	0.09	10.7 ± 20.2
sIL6-R, ng/ml	56.2 ± 19.3	57.8 ± 22.1	54.8 ± 16.8	0.85	0.83	47.3 ± 23.9
TJC	11.3 ± 6.9	12.28 ± 7.29	10.32 ± 6.41	0.06	0.04	5.6 ± 4.2
SJC	8.4 ± 5.8	8.80 ± 5.51	7.91 ± 6.04	0.13	0.37	3.7 ± 3.2
HAQ	1.1 ± 0.8	1.25 ± 0.75	1.01 ± 0.76	0.02	0.08	1.1 ± 0.6
DAS	3.4 ± 0.9	3.5 ± 1.0	3.3 ± 0.9	0.02	0.09	2.8 ± 0.8
SDAI	29.3 ± 14.3	30.8 ± 14.3	27.8 ± 14.3	0.09	0.36	15.8 ± 9.3
bDMARDs, n(%)	–	–	–	–	–	25 (46.3)
Chemerin, ng/ml	108.6 ± 52.2	120.7 ± 57.1	96.2 ± 43.6	0.002	0.01	53.7 ± 19.0
PEDF, μg/ml	13.1 ± 3.9	13.7 ± 3.9	12.5 ± 3.8	0.02	0.18	16.5 ± 3.2

*Values are mean ± standard deviation unless otherwise indicated. ERA, early rheumatoid arthritis; BMI, body mass index; ESR, erythrocyte sedimentation rate; CRP, C-reactive protein; DAS, disease activity score; ACPA, anti-citrullinated peptide antibodies; RF, rheumatoid factor; TJC, tender joint count; SJC, swollen joint count; HAQ, Health Assessment Questionnaire; SDAI, simplified disease activity index; bDMARDs, biological Disease Modifying Anti-Reumatic Drugs; PEDF, pigment epithelium-derived factor*.

# for Study population-1 and

## *for Study population-2*.

*P-value: Mann-Withney test overweight/obese patients vs. normal weight patients*.

P-value*: Mann-Withney test overweight/obese patients vs. normal weight patients corrected for age and sex.

*P-value < 0.05 were considered statistically significant*.

Moreover, to investigate whether specific molecules related to adipose tissue excess are associated with the inflammatory burden of RA, PEDF, and Chemerin plasma values were assessed in RA patients at disease onset compared to healthy subjects stratified by BMI category [30 healthy individuals, among whom 70% female, with a mean age of 50.0 years (*SD* = 9.6) and 12 (40.0%) overweight or obese].

PEDF and Chemerin plasma values were similar in ERA patients at diagnosis (PEDF: 13.1 ± 3.9 μg/ml, Chemerin: 108.6 ± 52.2 ng/ml) and controls (PEDF: 13.7 ± 2.4 μg/ml; *p* = *ns*; Chemerin: 95.2 ± 34.1 ng/ml; *p* = *ns*), while IL-6 plasma values were significantly higher in ERA patients than in controls (20.3 ± 34.5 pg/ml vs. 1.6 ± 3.8 pg/ml; *p* < 0.001). No significant difference was found between the sIL-6R plasma values at study entry comparing RA patients and controls (*p* = 0.34). Moreover, in ERA cohort, PEDF and Chemerin values directly correlated with age at the time of diagnosis (Supplemental Table 1).

However, higher plasma values of PEDF and Chemerin were observed in overweight/obese individuals than in normal weight ones (Figures 1B,C) and this difference remained significant only for Chemerin after age and sex adjustment (*p* = 0.01) (Table 1). Moreover, overweight/obese ERA patients showed higher IL-6 plasma values (26.8 ± 43.1 pg/ml) compared to normal weight (13.8 ± 21.3 pg/ml; *p* = 0.01) at baseline directly correlating with Chemerin plasma values (*R* = 0.25; *p* = 0.003). Considering sIL-6R in ERA cohort, overweight/obese ERA patients did not differ from normal weight ones at baseline (*p* = 0.85) with no significant correlation with Chemerin plasma values (*R* = −0.14; *p* = 0.36) at disease onset.

These data were confirmed by the positive correlation between PEDF, Chemerin and IL-6 plasma values and BMI (Supplemental Figure 1) (Supplemental Table 1). Optimal Chemerin cut-off value associated with BMI ≥ 25 Kg/m^2^ was 95.7 ng/ml with sensitivity and specificity of 60.0 and 60.9%, respectively. Optimal PEDF cut-off value associated with a BMI ≥ 25 Kg/m^2^ was 13.3 ug/ml with a sensitivity and specificity of 50.3 and 63.2%, respectively (Supplemental Figure 2).

### Chemerin but not PEDF plasma values are independently associated with baseline disease activity in ERA patients

In ERA patients (Study population-1), Chemerin but not PEDF plasma values positively correlated with disease activity scores (DAS: *R* = 0.33; *p* < 0.001; SDAI: *R* = 0.30; *p* < 0.001) and disability index (HAQ: *R* = 0.21; *p* = 0.01) (Supplemental Table 1 and Figure 1D).

In particular, ERA patients with Chemerin plasma values ≥ 95.7 ng/ml had more likely a moderate-high disease activity (46.6%) compared to RA patients with Chemerin plasma values < 95.7 ng/ml (27.6%; *p* = 0.01). On the contrary, no significant association was observed between PEDF plasma values and disease activity at diagnosis.

### Chemerin but not PEDF plasma levels values are independently associated with clinical outcome in ERA patients treated according to treat to target strategy

**Figure 2:**
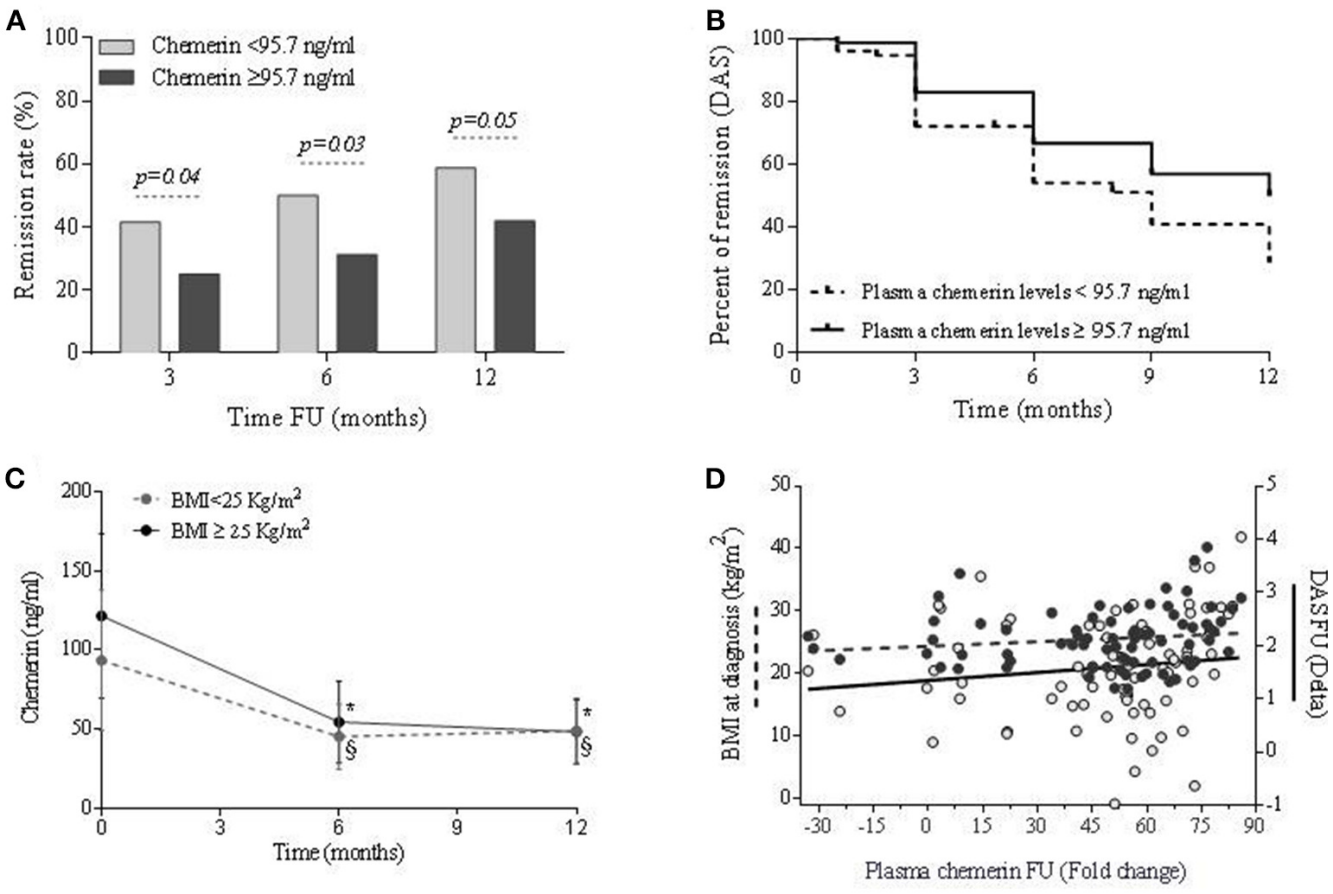
Association between chemerin plasma values and response to therapy over time in ERA patients treated according to a treat-to-target (T2T) strategy. **(A)** Association between Chemerin plasma values and percentage of remission (DAS < 1.6) at 3, 6, and 12 months of T2T treatment. **(B)** Kaplan-Meier survival curve in ERA patients achieving remission over 1 year stratified for the baseline Chemerin plasma values cut-off point (Log Rank test: χ^2^:8.72; *p* = 0.003; Breaslow-Wilcoxon text: χ^2^:7.24; *p* = 0.01). **(C)** Chemerin plasma values at baseline and during treat to target protocol (at 6 and 12 months) in ERA patients; *p < 0.05 vs. baseline in ERA patients with BMI ≥ 25 Kg/m^2^, §*p* < 0.05 vs. baseline in ERA patients with BMI < 25 Kg/m^2^; ^#^*p* < 0.05 for difference at different time points between BMI ≥ 25 Kg/m^2^ and BMI < 25 Kg/m^2^. **(D)** Correlation between changes in Chemerin plasma values, BMI at baseline and disease activity; Delta, (value at baseline - value at 12 months); Fold change, Delta/(value at baseline).

In the whole ERA cohort (Study population-1), DAS remission was achieved by 40.7 and 50.4% after 6 and 12 months follow-up, respectively, and SDAI remission status by 29.1% at 6 and 35.7% at 12 months follow-up, respectively. Overweight/obesity *per se* was not influencing remission achievement (Table 2). However, a higher and faster remission rate was reached by RA patients having Chemerin plasma levels < 95.7 ng/ml at baseline (Figures 2A,B), despite similar bDMARDs usage (Table 2), in addition to being VERA or having a high disease activity at the time of diagnosis (Table 2). On the contrary, high PEDF plasma levels values didn't influence treatment response (Table 2). The logistic regression analysis confirmed the independent association between therapy response at 12 months and Chemerin plasma values at baseline [OR(95%CIs):0.49(0.25–0.97)], together with being VERA [OR(95%CIs):2.05(1.00–4.23)], suggesting that low Chemerin plasma values is a predictor of remission (Table 2). This association was observed also using a stricter remission parameter as SDAI (28.6% of SDAI remission in ERA patients having Chemerin plasma values ≥95.7 ng/ml vs. 42.4% of SDAI remission in ERA patients having Chemerin plasma values < 95.7 ng/ml; *p* = 0.10). These findings suggest that a low Chemerin plasma values is a positive predictor of remission achievement in RA.

**Table 2:** Logistic regression analysis showing factors independently associated with DAS-remission (DAS < 1.6) at 6 and 12 months of follow-up in a cohort of ERA patients treated according to the T2T strategy.

	**DAS-remission (6 months FU)**		**DAS-remission (12 months FU)**	
**Variables**	Univariate analysis^*^ OR (95%CI)	**Logistic regression model OR (95%CI)**	Univariate analysis^*^ OR (95%CI)	**Logistic regression model OR (95%CI)**
Age, years	**0.97 (0.935–0.997)**	**0.97 (0.95–0.99)**	**0.98 (0.96–1.01)**	**0.99 (0.96–1.01)**
Sex (female)	0.65 (0.30–1.41)	–	0.55 (0.26–1.20)	–
VERA	**2.22 (1.08**–**4.55)**	**2.75 (1.26**–**5.98)**	**2.10 (1.03**–**4.28)**	**2.05 (1.00**–**4.23)**
Current Smokers, n. (%)	0.72 (0.33–1.58)	–	0.68 (0.32–1.44)	–
BMI ≥ 25 Kg/m^2^	0.65 (0.34–1.24)	–	0.75 (0.38–1.49)	–
AB positivity, n. (%)	1.23 (0.58–2.71)	–	1.27 (0.60–2.66)	–
DAS > 3.7 (active disease at baseline)	**0.33 (0.15**–**0.76)**	**0.37 (0.16**–**0.88)**	0.65 (0.32–1.35)	–
bDMARDs at 3 months FU	0.51 (0.13–2.05)	–	1.78 (0.49–6.42)	–
bDMARDs at 6 months FU	–		1.14 (0.39–3.35)	–
Chemerin ≥ 95.7 ng/ml	**0.47 (0.23**–**0.95)**	0.66 (0.31–1.43)	**0.48 (0.24**–**0.94)**	**0.49 (0.25**–**0.97)**
PEDF ≥ 13.3 μg/ml	0.84 (0.42–1.67)	–	1.03 (0.53–2.02)	–

*VERA, Very Early Rheumatoid Arthritis patients; BMI, Body Mass Index; AB, Autoantibodies; DAS, Disease Activity Score; bDMARDs, biological Disease Modifying Anti-Rheumatic Drugs; FU, follow-up; PEDF, Pigment Epithelium Derived Factor; OR, Odds Ratio; CI, Confidential Interval; FU, Follow-up*.

* *Mann–Whitney U-test or χ2 test. Bold, p-value < 0.05*.

As previously stated, a significant disease activity reduction at 6 and 12 months was observed in both normal weight and overweight-obese ERA patients. Similarly, Chemerin plasma values significantly decreased over time reaching comparable levels already after 6 months in overweight/obese and normal weight RA patients, showing a higher rate of Chemerin plasma values reduction in overweight/obese compared to normal weight ERA patients (Figure 2C). On the contrary, PEDF plasma values remained unchanged during the follow-up (data not shown). The entity of Chemerin plasma values reduction was found tightly related to the initial BMI (*R* = 0.26; *p* = 0.02) and directly correlated with the DAS value reduction (*R* = 0.24; *p* = 0.03) in ERA patients (Figure 2D). Furthermore, this finding was confirmed by the direct correlation between Chemerin plasma values reduction and the delta SDAI (*R* = 0.28; *p* = 0.02). Therefore, in ERA patients changes of Chemerin plasma values but not of PEDF, throughout the T2T treatment, is associated with baseline BMI and mirrors the reduction of disease activity despite stable body weight.

### Adipose tissue of obese RA patients shows different expression of adipokines, IL-6 and their receptors based on disease activity

To reveal whether WAT of patients with RA shows different gene expression profile based on disease itself and/or fat-related inflammation, we investigated the quantitative expression of *RARRES2, CMKLR1, SERPINF1, IL6*, and *IL6-R* in WAT of obese RA (BMI: 35.5 ± 4.1 Kg/m^2^) and OA (BMI: 34.8 ± 3.2).

**Figure 3:**
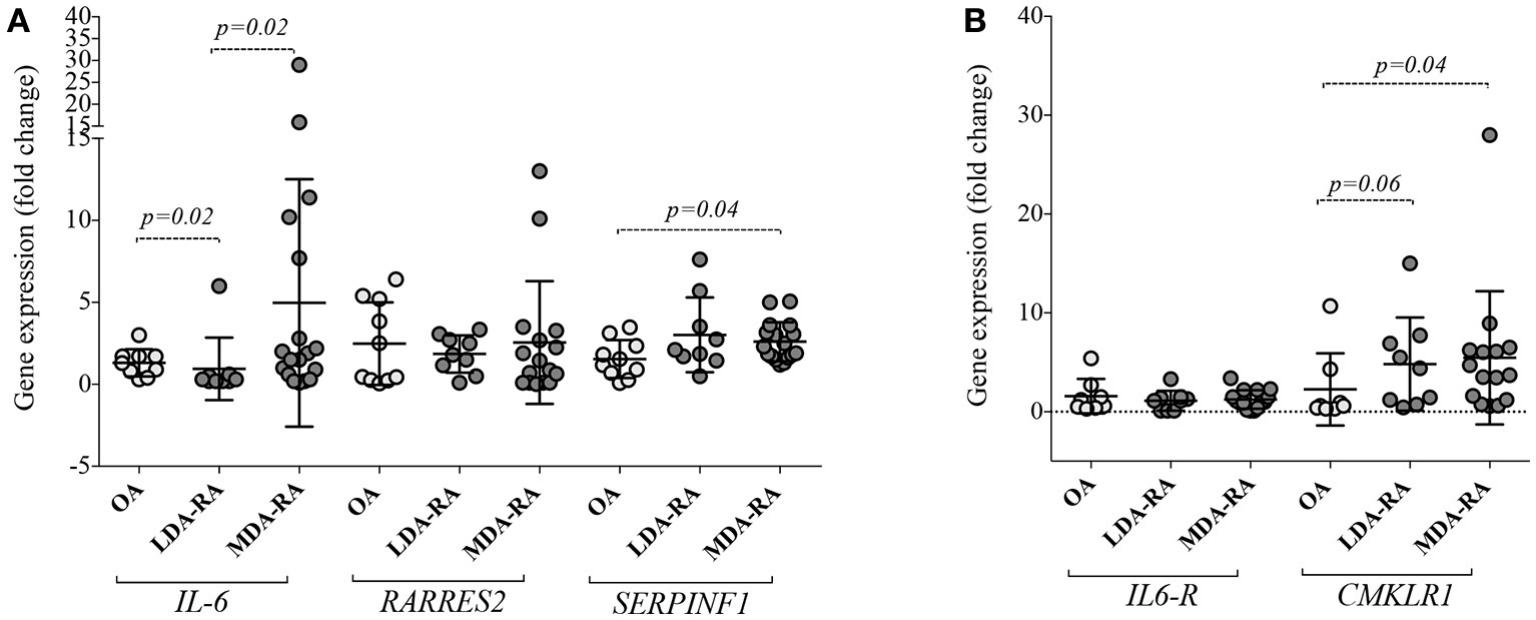
Chemerin, PEDF, IL6 and their receptors expression in White Adipose Tissue (WAT) of obese RA patients with low-moderate disease activity and in obese OA. **(A)** *IL6, RARRES2*, and *SERPINF1* expression and **(B)** *CMKLR1* and *IL6-R* were determined in abdominal WAT from obese RA (*n* = 27) and obese OA (*n* = 10). Levels of target genes mRNA expression were determined, after normalization with GAPDH values, using the 2−^Δ^ΔC_T_ method. Horizontal lines indicate mean values with SD. RA, Rheumatoid Arthritis; OA, Osteoarthritis; LDA, Low disease activity (1.6 < DAS < 2.4); MDA, Moderate disease activity (2.4 < DAS < 3.7); *IL6*, Interleukin 6; *RARRES2*, retinoic acid receptor responder protein 2; *CMKLR1*, Chemokine like receptor 1; *IL6-R*, interleukin-6 receptor.

In particular, we found that *SERPINF1* was highly expressed in WAT of RA (2.7 ± 1.6 fold change) than OA patients (1.5 ± 1.2 fold change; *p* = 0.03), mainly in RA with moderate disease activity (MDA) (Figure 3A). No differences were seen for *RARRES2* and *IL6* expression in WAT between the whole obese RA and OA cohorts. However, stratifying patients based on the disease activity, WAT from MDA RA showed higher *IL6* expression compared to RA patients with low disease activity (LDA) (Figure 3A). Increased expression of *CMKLR1* was observed in WAT of obese RA (7.2 ± 12.1 fold change) compared to OA (2.3 ± 3.7 fold change; *p* = 0.02), particularly in MDA RA (7.4 ± 12.1; *p* = 0.04) (Figure 3B). Finally, no correlation was found between adipokines and their receptors expression in WAT and Chemerin, PEDF or IL6 plasma values or disease activity (i.e., DAS) in obese RA patients (data not shown).

### Weight loss induces chemerin and PEDF plasma values reduction, influencing the remission achievement in overweight/obese RA patients

We enrolled 54 overweight/obese RA patients with low-moderate disease activity, despite a treat to target strategy treatment, undergoing low calories diet for at least 6 months (Study population-2) (Table 1). During the follow-up, the mean BMI reduction was 2.5 ± 1.8 Kg/m^2^ (corresponding to 7.4 ± 5.7% of initial BMI) at 6 months and 3.1 ± 2.4 Kg/m^2^ (corresponding to 8.9 ± 6.8% of initial BMI) at 12 months of diet, respectively. However, 7 (13.0%) RA patients did not show any change in BMI during the follow-up.

**Figure 4:**
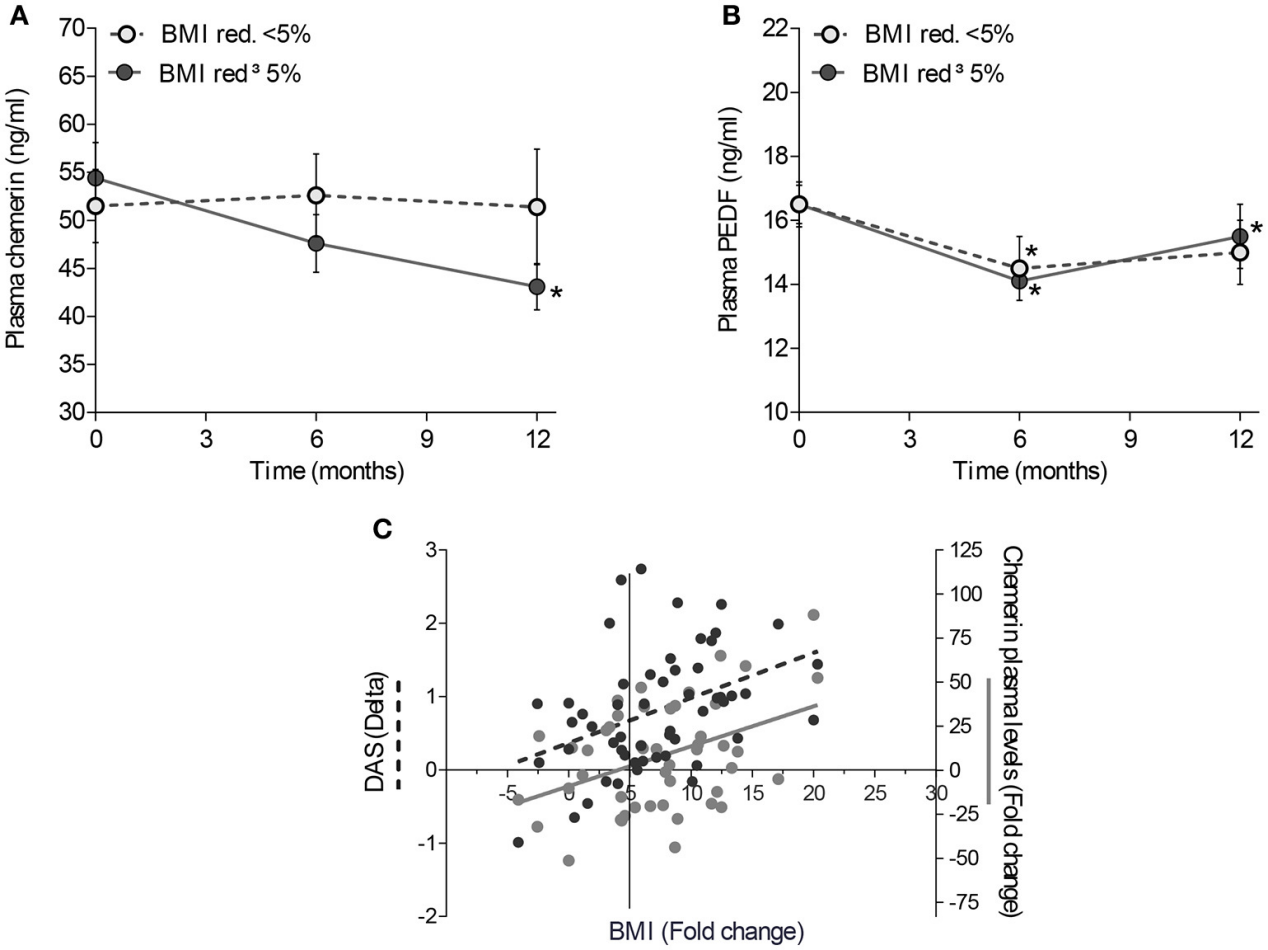
Effect of weight loss on Chemerin and PEDF plasma values. Mean changes in Chemerin **(A)** and PEDF **(B)** plasma values from baseline in overweight/obese RA patients with a low-moderate disease activity undergoing scheduled dietetic regimen aiming at BMI reduction. RA patients were divided based on the percentage of reduction of BMI (< or ≥ 5%); *: *p* ≤ 0.05. **(C)** Correlation between changes in BMI, plasma values of Chemerin and disease activity; Delta, (value at baseline - value at 6 months); Fold change, Delta/(value at baseline).

To clarify whether weight loss was directly associated with adipokines modulation in RA patients, Chemerin and PEDF plasma values were tested at baseline and after a low-calories diet (Study population-2). A significant reduction of Chemerin and PEDF plasma values was observed since the 6TH month of low-calories diet, in patients achieving ≥5% BMI reduction (Figures 4A,B). Moreover, a direct correlation between the BMI fold change (baseline/6 months) and Chemerin plasma values fold change was found (*R* = 0.30; *p* = 0.04) (Figure 4C), whereas there was no correlation with PEDF plasma values fold change. Finally, RA patients achieving a decrease of BMI ≥ 5% at 6 months showed a significant reduction of sIL-6R plasma values at 12 months follow-up (47.9 ng/ml at baseline vs. 40.4 ng/ml at 12 months follow-up; Wilcoxon test: *p* = 0.04), not observed in RA patients with BMI reduction < 5% during the same follow-up period (*p* = 0.10). IL-6 plasma values were similar in the two subgroups (data not shown).

RA patients with low-moderate disease activity (Study population-2) reducing ≥5% the baseline BMI after 6 months, showed a significant reduction of disease activity after 6 and 12 months, without any pharmacological change (Supplemental Figure 3):34 RA patients reaching ≥5% BMI reduction after 6 months and 19 with ≥10% BMI reduction after 12 months, obtained higher DAS remission rates than patients with a less significant BMI reduction. In addition, 39.4% of RA patients experiencing BMI decrease ≥5% achieved DAS remission at 6 months follow-up compared to 10.0% of RA patients achieving BMI decrease < 5% (*p* = 0.02), whereas 55.6% RA patients experiencing BMI decrease ≥10% achieved DAS remission at 12 months follow-up compared to 15.0% of RA patients achieving BMI decrease < 10% (*p* = 0.01). The effect of weight reduction on disease activity was already seen at 6 months, as confirmed by the positive correlation between the BMI fold change (baseline/6 months) and delta DAS (*R* = 0.45; *p* = 0.001) as well as delta SDAI (*R* = 0.49; *p* < 0.001).

## Discussion

Over the last years, understanding the importance of overweight and obesity during RA disease course, from the disease onset through its progression and outcome has increased and improved. To date, adipose tissue arose as a dynamic endocrine organ that releases bioactive substances secreted by adipocytes and adipose tissue resident macrophages shared with chronic inflammatory diseases as RA (30). Most of these molecules show pro-inflammatory properties accounting for a low-grade systemic inflammation. Among them, Chemerin appears to be a crucial inflammatory mediator in RA, correlating with disease activity rather than obesity (17, 31) whereas PEDF plays a role in the promotion of inflammation (9, 12).

Our data show that Chemerin plasma values positively correlate with BMI and disease activity in RA patients at diagnosis. Moreover, a low calories Mediterranean diet induces weight loss in an important percentage of overweight/obese RA patients, increasing the number of patients achieving disease remission. In fact, we observed, for the first time, that a significant percentage of RA patients with low-moderate disease activity can be led to clinical remission without any treatment change by loosing at least 5% of baseline BMI which is a commonly recommended therapeutic target for obese people leading to profound health benefits (28, 32). In particular, effective dietary intervention significantly affects direct and indirect adipose tissue-related inflammatory markers, as Chemerin and sIL-6R respectively, possibly by positively reducing the recruitment of adipose tissue macrophages as the underlying features of meta-inflammation (33, 34). These data underline the importance of fat-derived inflammatory mediators on disease activity in obese RA patients.

Since the direct link between high BMI and disease activity at RA onset is still a matter of debate, we investigated the clinical features of RA patients at disease onset stratified according to their BMI category. Interestingly, we found that, at disease onset, overweight/obese RA patients had higher disease activity compared to normal weight ones. Moreover, analyzing the influence of body weight excess in terms of adipose tissue derived inflammatory markers on the remission rate achievement in ERA patients treated with the T2T scheme, high Chemerin plasma values at diagnosis were negatively associated to the achievement of good disease control after the T2T scheme. The impact of obesity on clinical outcome was previously investigated in long standing RA (8) despite the controversial indirect link between obesity and bone erosiveness (18, 35, 36). Considering adipose tissue derived adipokines, Ha et al previously found an indirect correlation between BMI and Chemerin plasma values evaluated in a limited RA cohort, stating that Chemerin plasma values could be associated with systemic inflammation rather than obesity in RA. However, this latter cohort was made of already treated patients with long standing disease and no data were reported about Chemerin plasma values at disease onset (17). It should be considered that, in the general population, Chemerin plasma values directly correlate not only with the obesity status, in terms of BMI category, total body fat percentage, waist circumference and total lipids asset but even with multiple markers of chronic inflammation (i.e., CRP, SAA, IL-6, and sTNFRII) suggesting that it may induce extensive inflammatory processes in various chronic inflammatory disorders (37).

In our ERA cohort, a significant association between Chemerin and PEDF plasma values and parameters of systemic inflammation was found at the time of diagnosis, even though only Chemerin plasma values positively correlated with disease activity. These data mirror cytokines' behavior, as IL-6, which is a crucial player in the inflammatory cascade at the earliest stages of RA (38), suggesting that Chemerin may play a role in RA, mainly in patients with high BMI. Interestingly in our study, higher Chemerin plasma values at disease onset, but not the overweight-obesity status nor higher PEDF plasma values, was associated with remission achievement, identifying Chemerin as a biomarker of metaflammation and as a modifiable risk factor associated with RA treatment response (16, 39).

In animal models, it is well documented that high-fat diet induces a low-grade chronic inflammation called metaflammation (40) leading to the production of pro-inflammatory cytokines as IL-1, IL-6, and Tumor Necrosis Factor (41–43), all over-expressed at the early phases of RA (38). These pro-inflammatory cytokines affect synovial and adipose tissues causing inflammation that promotes the recruitment and activation of immune cells within these target tissues and the amplification of the inflammatory loop in RA (44, 45). Based on this, we investigated the gene profile of IL-6, Chemerin/ChemR23 and PEDF in abdominal adipose tissue derived from obese RA patients, finding a higher expression of IL-6, ChemR23, and PEDF in WAT of obese RA patients with MDA than in obese OA. These findings suggest that the gene expression profile of pro-inflammatory mediators in WAT is tightly related to the inflammatory status mirroring the disease activity phases in obese RA. Moreover, studies on animal models revealed that obesity influences the degree of inflammation in another biological compartment involved in RA pathogenesis, as synovial tissue, which seems to be affected by high fat diet mainly at the earliest disease phases in terms of synovial hyperplasia and inflammatory cells infiltrates with no significant differences between obese and lean animals after disease establishment (46).

In conclusion, PEDF and Chemerin can be biomarkers of obesity and metaflammation in RA patients respectively. In particular, Chemerin seems to be linked to RA disease activity and treatment response, supporting its dual role in mirroring inflammation and metabolism and providing a link between chronic inflammation and obesity. Based on that, a BMI reduction of at least 5%, mirrored by Chemerin modulation, allows the achievement of a better disease control without changing RA treatment.

## Author contributions

BT, GF, and EG conceived the study. MRG, SA, LP, ALF, BA, and MRM collected clinical data. BT, MRG, SA, LP, and CD performed experiments. BT, MRG, SA, and LP performed statistical analysis. BT, MRG, SA, LP, ALF, GF, and EG drafted and revised the manuscript. All authors read and approved the final manuscript.

### Conflict of interest statement

The authors declare that the research was conducted in the absence of any commercial or financial relationships that could be construed as a potential conflict of interest.
