# Supplementary material for: Chemerin and PEDF Are Metaflammation-Related Biomarkers of Disease Activity and Obesity in Rheumatoid Arthritis
Source: Front Med (Lausanne). 2018 Aug 3;5:207. doi: 10.3389/fmed.2018.00207 (PMC6085446; doi:10.3389/fmed.2018.00207)

Supplementary Information

CHEMERIN AND PEDF ARE METAFLAMMATION-RELATED BIOMARKERS OF DISEASE ACTIVITY AND OBESITY IN RHEUMATOID ARTHRITIS

Tolusso Barbara[1], Gigante Maria Rita[1], Alivernini Stefano[1], Petricca Luca[1], Fedele Anna Laura[1], Di Mario Clara[1], Aquilanti Barbara[2], Magurano Maria Rosaria[3], Ferraccioli Gianfranco[1], Gremese Elisa[1,*].

1. Division of Rheumatology, Fondazione Policlinico Universitario A. Gemelli, Catholic University of the Sacred Heart, Rome, Italy
2. Service of Dietary and Human Nutrition, Fondazione Policlinico Universitario A. Gemelli, Catholic University of the Sacred Heart, Rome, Italy
3. Service of Psychology and Psychotherapy, Fondazione Policlinico Universitario A. Gemelli , Catholic University of the Sacred Heart, Rome, Italy

Supplemental Figure 1. PEDF and Chemerin plasma levels in ERA patients correlate with BMI at diagnosis. Association between PEDF and Chemerin plasma levels, measured by ELISA, and BMI in ERA patients at diagnosis.

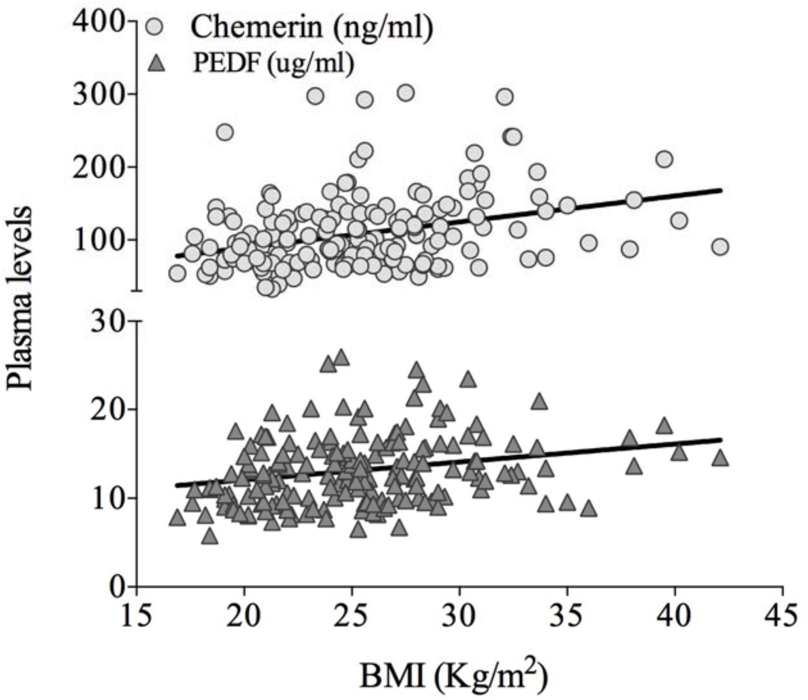

Supplement: Supplementary file 1 [file Image_1.pdf]
