# Supplementary material for: Chemerin and PEDF Are Metaflammation-Related Biomarkers of Disease Activity and Obesity in Rheumatoid Arthritis
Source: Front Med (Lausanne). 2018 Aug 3;5:207. doi: 10.3389/fmed.2018.00207 (PMC6085446; doi:10.3389/fmed.2018.00207)

Supplemental Figure 2. Receiver operating characteristic (ROC) analysis. Capacity of PEDF and Chemerin plasma levels to discriminate between ERA patients with or without an overweight/obese status. The area under the curve (AUC) was 0.636 (95%CI=0.554-0.717, p=0.002) for Chemerin and 0.602 (95%CI=0.519-0.685, p=0.02) for PEDF.

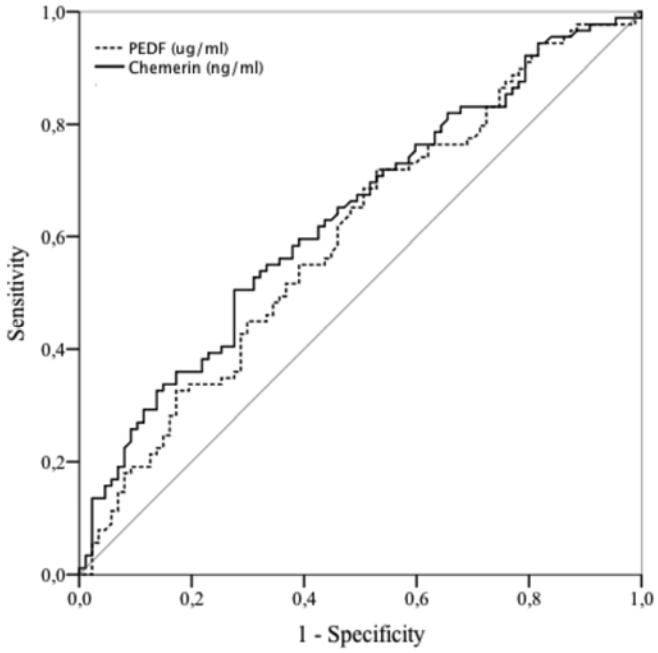

Supplement: Supplementary file 2 [file Image_2.pdf]
