# Supplementary material for: Chemerin and PEDF Are Metaflammation-Related Biomarkers of Disease Activity and Obesity in Rheumatoid Arthritis
Source: Front Med (Lausanne). 2018 Aug 3;5:207. doi: 10.3389/fmed.2018.00207 (PMC6085446; doi:10.3389/fmed.2018.00207)

Supplemental Figure 3. Mean changes in disease activity from baseline in overweight/obese RA patients with a low-moderate disease activity undergoing a scheduled dietetic regimen aiming to weight loss. Patients were divided based on the percentage of reduction of BMI (< or ≥ 5%); *: *p≤0.05* and **: *p≤0.01*.

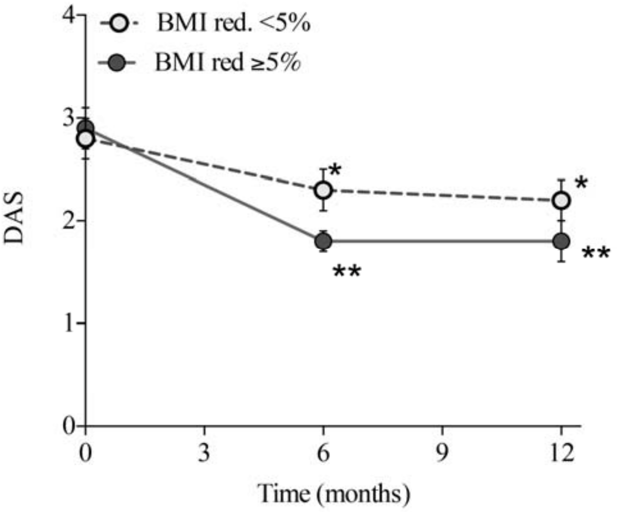

Supplement: Supplementary file 3 [file Image_3.pdf]
