# Supplementary material for: Chemerin and PEDF Are Metaflammation-Related Biomarkers of Disease Activity and Obesity in Rheumatoid Arthritis
Source: Front Med (Lausanne). 2018 Aug 3;5:207. doi: 10.3389/fmed.2018.00207 (PMC6085446; doi:10.3389/fmed.2018.00207)
Supplement: Supplementary file 4 [file Table_1.doc]

**Supplementary Table 1. Correlation analysis between BMI, circulating levels of Chemerin and PEDF with ERA demographic and clinical characteristics at the time of diagnosis.**

| **Variables** | **BMI**  (Kg/m^2^) | | **Chemerin**  (ng/ml) | | **PEDF**  (µg/ml) | |
| --- | --- | --- | --- | --- | --- | --- |
|  | r | *p* | r | *p* | r | *p* |
| **Age, years** | 0.24 | ***0.001*** | 0.30 | ***<0.001*** | 0.33 | ***<0.001*** |
| **Symptom’s duration, months** | -0.13 | *0.08* | -0.12 | *0.12* | 0.02 | *0.77* |
| **BMI, Kg/m^2^** | - | *-* | - | *-* | - | *-* |
| **ESR, mm/1^hour** | 0.18 | ***0.01*** | 0.37 | ***<0.001*** | 0.17 | ***0.02*** |
| **CRP, mg/l** | 0.22 | ***0.004*** | 0.32 | ***<0.001*** | 0.21 | ***0.01*** |
| **IL6,** | 0.22 | ***0.01*** | 0.25 | ***0.003*** | 0.07 | *0.43* |
| **TJC** | 0.07 | *0.35* | 0.21 | ***0.01*** | 0.003 | *0.97* |
| **SJC** | 0.08 | *0.27* | 0.25 | ***0.001*** | 0.01 | *0.90* |
| **VAS** | 0.06 | *0.42* | 0.22 | ***0.003*** | 0.06 | *0.44* |
| **HAQ** | 0.19 | ***0.01*** | 0.21 | ***0.01*** | 0.11 | *0.13* |
| **PGA** | -0.44 | *0.56* | 0.12 | 0.12 | 0.02 | *0.84* |
| **PhGA** | -0.001 | *0.99* | 0.16 | ***0.04*** | -0.03 | *0.70* |
| **DAS** | 0.15 | ***0.04*** | 0.33 | ***<0.001*** | 0.06 | *0.43* |
| **SDAI** | 0.11 | *0.15* | 0.30 | ***<0.001*** | 0.12 | *0.15* |
| **Cholesterol, mg/dL** | 0.05 | *0.53* | -0.03 | *0.73* | 0.06 | *0.41* |
| **HDL, mg/dL** | -0.25 | ***0.02*** | -0.25 | ***0.002*** | -0.09 | *0.29* |
| **Triglycerides, mg/dL** | 0.24 | ***0.003*** | 0.32 | ***<0.001*** | 0.23 | ***0.01*** |
| **Chemerin, ng/ml** | 0.31 | ***<0.001*** | - | ***-*** | - | *-* |
| **PEDF, µg/ml** | 0.29 | ***<0.001*** | 0.26 | ***<0.001*** | - | *-* |

*Values are mean ± standard deviation unless otherwise indicated.* ***BMI****: Body Mass Index;* ***ESR****: Erytrocyte sedimentation rate;* **CRP**: C-Reactive Protein; **IL-6**: Interleukin 6; **TJC**: Tender Joint Count; **SJC**: Swollen Joint Count; **VAS**: Visual Analogue Scale; **HAQ**: Health Assessment Questionnaire; **PGA**: Patient Global Assessment; **PhGA**: Physician Global Assessment; **DAS**: Disease Activity Score; **PEDF**:pigment epithelium derived factor.
